# Pacemaker-Induced Superior Vena Cava Syndrome

**DOI:** 10.7759/cureus.75758

**Published:** 2024-12-15

**Authors:** Michael N Zarrella, Kolu Wynne, Basel Saadeh, Gregory Gersten

**Affiliations:** 1 Internal Medicine, St Mary's Hospital, Waterbury, USA; 2 Interventional Radiology, St Mary's Hospital, Waterbury, USA

**Keywords:** angiography and angioplasty, balloon angiography, permanent pacemaker (ppm) complication, superior vena cava (svc) syndrome, tachy-brady syndrome

## Abstract

Superior vena cava (SVC) syndrome is a result of impaired blood flow from the SVC to the right atrium, leading to venous congestion in the head and neck. It can be caused by clotting disorders or compressive tumors of the head and neck but has become more prevalent in the setting of implantable devices such as pacemakers. As such, managing these patients can present challenges for physicians who have to account for SVC syndrome as well as their underlying condition requiring an implantable cardiac device. Our case represents one such patient who developed SVC syndrome as a result of pacemaker lead-related formation that was treated with both invasive and noninvasive therapy. This presentation highlights the successful management of SVC syndrome in a patient with sick sinus syndrome. It also demonstrates the efficacy of balloon angioplasty in managing this particular type of SVC syndrome, as well as exemplifying the use of leadless pacemaker devices as a means of long-term prevention.

## Introduction

Superior vena cava (SVC) syndrome results from an obstruction of blood flow through the SVC caused by either direct invasion of a tumor into the SVC, external compression on the vessel from surrounding structures, or foreign body-related inflammatory stricture formation. SVC syndrome typically presents with symptoms signifying underlying thoracic central venous obstruction, including swelling of the head and neck, shortness of breath, chest pain, and neurologic manifestations [1].

While malignancy accounts for the majority of cases of SVC syndrome, the overall incidence of device-related SVC syndrome now accounts for 20-40% of cases due to the increasing use of intravascular devices [2]. In particular, devices placed within the thoracic central venous system are one of the most common causes of thoracic central venous obstruction (TCVO), which is primarily related to devices positioned within or traversing through the subclavian veins. Trauma from the initial device insertion can damage the endothelium resulting in stenosis, obstruction, and scar tissue formation within the vein [3].

## Case presentation

A 56-year-old woman presented with a chief complaint of head and neck swelling that has been progressively worsening over the past three months. The patient expressed concern regarding the prominence of the veins in her neck, face, and chest, with overlying erythema. She denied having recent headaches, cough, or difficulty breathing.

The patient's past medical history is significant for sick sinus syndrome, status post dual-chamber pacemaker, which was placed three years before this admission.

On physical exam, the patient had a temperature of 97.4 degrees Fahrenheit, heart rate of 82 beats per minute, blood pressure of 121/77 mmHg, respiratory rate of 16, and oxygen saturation of 100% on room air. The head and neck exam was notable for substantial head and neck edema associated with erythematous skin, prominent bilateral jugular venous distension extending to the angle of the mandible when sitting upright, and distended collateral veins throughout the superior chest wall. The cardiovascular exam showed a normal S1 and S2, regular rate, and rhythm without murmurs, rubs, or gallops. The respiratory exam showed clear lungs bilaterally, without wheezing, rales, or rhonchi. Peripheral extremities show 2+ radial, dorsalis pedis, and posterior tibial pulses bilaterally, without lower extremity edema.

Laboratory data revealed grossly unremarkable complete metabolic panel and complete blood count. High-sensitivity troponin, B-type natriuretic peptide, prothrombin time, and international normalized ratio were all within normal limits.

Imaging studies revealed an electrocardiogram (ECG) showing normal sinus rhythm with first-degree atrioventricular (AV) block. Chest X-ray (CXR) showed no focal consolidations, effusions, or pneumothorax, with a left-sided pacemaker in place, otherwise grossly unremarkable.

**Figure 1:**
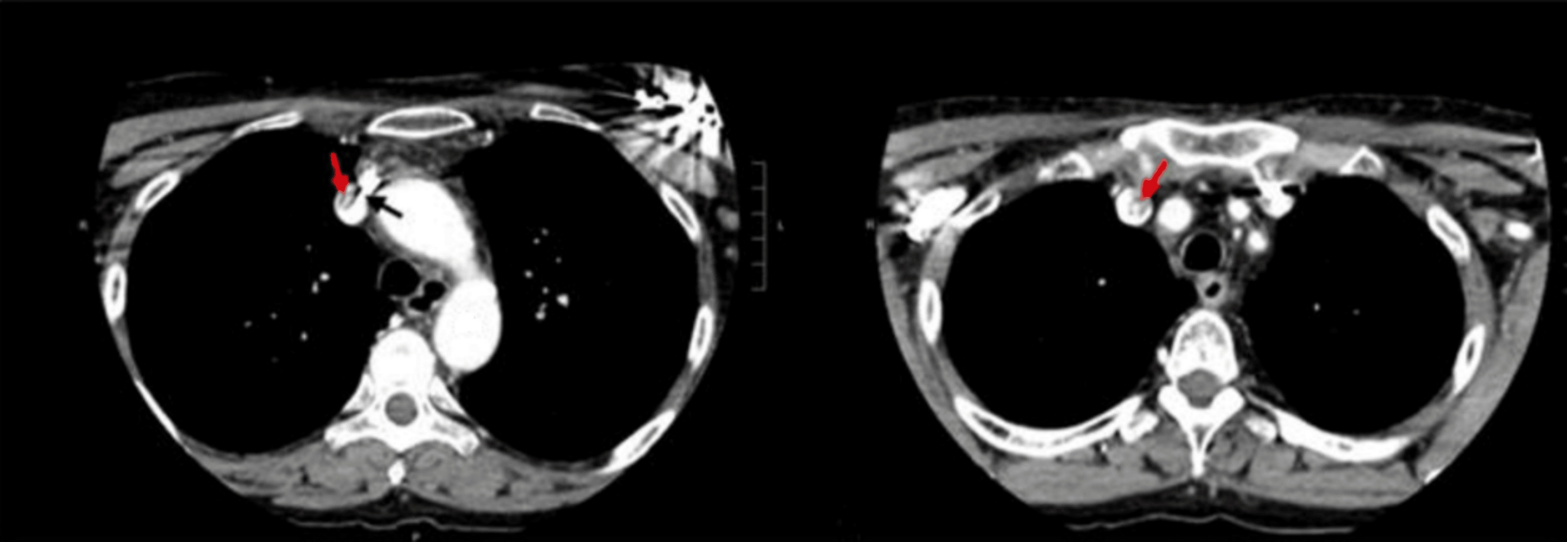
Axial CT neck with IV contrast, demonstrating stricture formation (red arrow) within the superior vena cava, with adjacent pacemaker lead (black arrow). CT: computed tomography.

**Figure 2:**
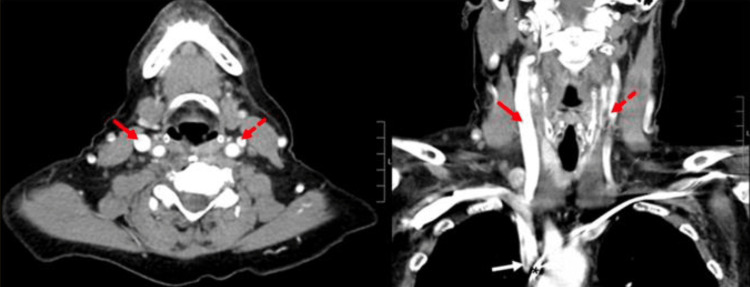
Axial (A) and coronal (B) CT neck with IV contrast, showcasing unilateral engorgement of the right internal jugular vein (solid red arrows) in comparison with the left internal jugular vein (dashed red arrows) secondary to stricture formation (solid white arrow) seen in the mid superior vena cava and right brachiocephalic vein adjacent to pacemaker leads (black asterisk). CT: computed tomography.

Computed tomography angiography (CTA) revealed evidence of SVC syndrome, with unilateral engorgement of the right internal jugular vein compared to the left, and stricture formation adjacent to the pacemaker leads, as shown in Figures 1 and 2.

**Figure 3:**
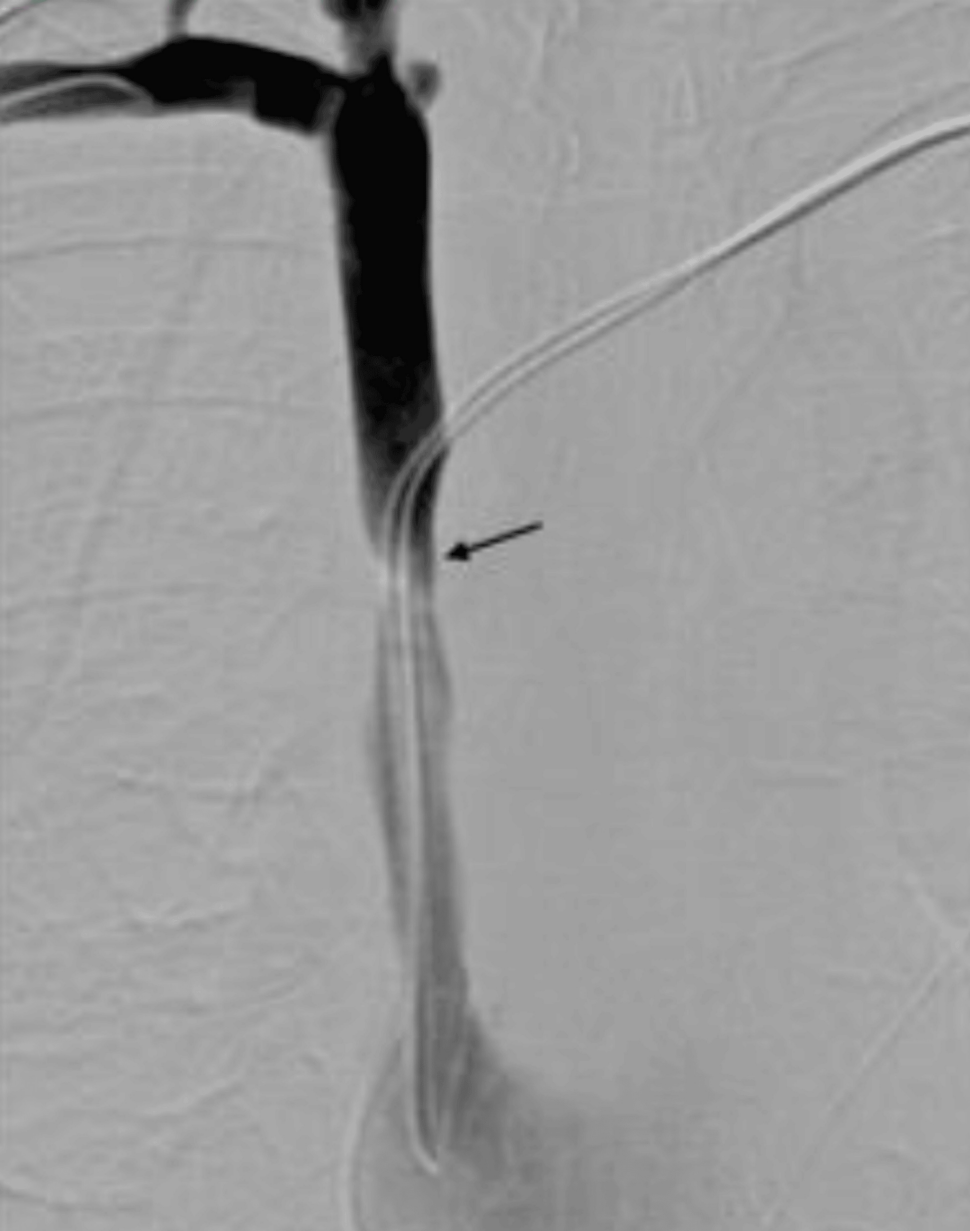
Digital subtraction angiography. Intraprocedural imaging of SVC demonstrating a severe web-like stricture in the mid-SVC (black arrow), with moderate upstream dilation of the SVC. SVC: superior vena cava.

A follow-up superior venacavagram demonstrated a severe weblike stricture in the mid-SVC surrounding the pacemaker leads with upstream dilation of the SVC and contrast refluxing into the internal jugular vein as well as the chest wall venous collaterals. Progressive angioplasty was performed successfully, which demonstrated patency and improvement in collateral flow, as shown in Figure 3.

Post-procedurally, patient was evaluated three months later and had mild recurrence of symptoms, which resulted in repeat balloon angiography. Additional follow-up at six months resulted in the removal of her implantable pacemaker, replacing it with a leadless pacemaker device to prevent further recurrence. As of the device exchange, the patient remains symptom-free.

## Discussion

The history of presenting illness is particularly important as it pertains to the potential causes of a patient’s SVC syndrome. SVC syndrome typically occurs most commonly due to etiologies categorized as either malignant or benign causes [1,4]. Malignant causes of SVC syndrome are most commonly related to non-small cell (50%), small cell (25%), and the remaining variable causes, including lymphoma, thymoma, and mediastinal lymph node metastasis [4]. The most common benign causes of SVC syndrome include pacemakers, defibrillators, and indwelling central venous catheters [4,5].

Management of SVC syndrome is entirely based on the etiology, which stresses the importance of history and workup. SVC syndrome caused by an implantable pacemaker or other implantable cardiac device (ICD) focuses on addressing the underlying device [1]. As it currently stands, there are limited guidelines to provide physicians with appropriate treatment or prevention. As demonstrated in this case, utilizing balloon angiography is a safe and effective way of managing SVC syndrome. Other cases involving similar etiology have also demonstrated success using balloon angioplasty with stenting [6], whereas others have had success with anticoagulation therapy alone [7]. One small study involving 28 patients with pacemaker- and ICD-induced SVC syndrome evaluated the use of transluminal balloon angioplasty as management and found that, over 24 months, patients underwent an average of at least two procedures per year to maintain patency [8]. The challenge in preventing symptomatic recurrence in this patient population appears to vary on a case-by-case basis.

Leadless pacemaker implantation may be a successful way for long-term prevention, as used in this case, but it is not without its own limitations. While leadless pacing can be used in the management of sick sinus syndrome, it is important to note that leadless devices can only pace the right ventricle and may not be ideal for patients who require more frequent pacing, which would otherwise be provided by the addition of atrial pacing [9].

## Conclusions

Pacemaker-induced SVC syndrome is a less common cause of the clinical syndrome, but it should be considered in patients with a relevant history. While guideline-directed management remains fairly new and undefined, several case reports and small studies have demonstrated success with the use of balloon angioplasty, although recurrence rates are considerable. Long-term outcomes garner the consideration of pacemaker removal and replacement with newer leadless intracardiac devices as more effective management. Due to limitations of leadless pacing, long-term management under the guidance of an electrophysiologist is recommended for these patients.
